# Early gestational maternal low-protein diet diminishes hepatic response to fasting in young adult male mice

**DOI:** 10.1038/s41598-017-10380-4

**Published:** 2017-08-29

**Authors:** Noriko Sato, Katsuko Sudo, Masayo Mori, Chihiro Imai, Masaaki Muramatsu, Masahiro Sugimoto

**Affiliations:** 1 Department of Epigenetic Epidemiology/Molecular Epidemiology, Medical Research Institute, Tokyo Medical and Dental University, 1-5-45, Yushima, Bunkyo-ku Tokyo, 113-8510 Japan; 2 Animal Research Center, Tokyo Medical University, 6-1-1, Shinjyuku, Shinjyuku-ku Tokyo, 160-0022 Japan; 3 Institute for Advanced Biosciences, Keio University, Mizukami, Kakuganji, Tsuruoka Yamagata, 997-0052 Japan; 4 AMED-CREST, AMED, 1-7-1 Otemachi, Chiyoda-Ku, Tokyo, 100-0004 Japan

## Abstract

Maternal low-protein (MLP) diet can lead to hepatic steatosis, which only develops with ageing. It is still unclear whether the young offspring show any signs of past exposure to prenatal adverse conditions. We hypothesized that early nutritional insult would first affect the dynamic responsiveness to nutritional challenges rather than the static state. We analyzed the transcriptome and metabolome profiles of the hepatic response to fasting/refeeding in young male mice offspring to identify changes induced by early gestational MLP diet. Restricted MLP exposure strictly to early gestation was achieved by the embryo transfer method. As a result, the fasting-induced upregulation of genes related to long-chain fatty acid metabolism and of stress response genes related to protein folding were significantly diminished in MLP pups. Lipid profiling after fasting showed that the hepatic signature of triacylglycerols was shifted to longer acyl-chains and higher saturation by the MLP diet. Bioinformatic analyses suggested that these phenomenological changes may be partially linked to the peroxisome proliferator activated receptor α (PPARα) pathway. Taken together, early gestational MLP diet affected the hepatic dynamic response to nutritional stress in seemingly healthy young offspring, accompanied with partial deterioration of PPARα action.

## Introduction

Human epidemiological studies have shown that early embryonic or fetal exposure to suboptimal nutrition raises the risk of obesity, metabolic disorder, and cardiovascular disease in later life^1, 2^. Animal studies have provided further evidence that maternal low-protein (MLP) diet amplifies age-associated changes towards dyslipidemia, hepatic steatosis, and shortened lifespan^3–5^. Erhuma *et al*. compared the effects of four different MLP exposure periods (early-, mid-, late-, and throughout gestation), exploring the possibility that disease liability is established during a specific critical window during the prenatal period^6^. They found, however, that MLP generates a broadly similar phenotype regardless of the timing of the insult^6^. Why early gestational MLP diet, independently of uteroplacental insufficiency, programs the development of a fatty liver remains unresolved. The early gestational period is of special interest, because it is the critical developmental window of epigenetic dynamics accompanying cell fate decision and differentiation. Maternal nutritional state and physique in early gestation correlate well with epigenetic variation, body composition diversity, and overall predisposition to adult disease in the offspring^1, 7–10^. To dissect the effects of early gestational insult in the offspring, it is important to refine the experimental procedures and strictly limit the exposure time in animal experiments.

Hepatic steatosis, dyslipidemia, and metabolic disorders are multifactorial diseases that develop over a long period of time. The risk exposures in early life gradually alter bodily function and eventually result in diseases at older ages^11, 12^. However, the precise mechanism by which metabolic homeostasis becomes disrupted during the pre-symptomatic state is not known. To maintain systemic metabolic homeostasis, an hepatic response to fasting/refeeding is crucial^13^. Therefore, dysregulation of the hepatic fasting response caused by prenatal exposure, if any, could be a trigger for a future metabolic disorder. From another point of view, prior to the onset of overt diseases, plasma metabolite profiles often become changed. As predictive markers of diabetes, plasma branched-chain amino acids and lipid profiling recently attracted attention^14, 15^. In this context, it is of interest to investigate the existence of pre-symptomatic metabolomic markers in the liver that are indicative of past exposure to prenatal adverse conditions.

In this study, we tested whether early gestational MLP diet affects hepatic metabolism even in seemingly healthy young offspring. To uncover differences caused by early gestational MLP diet in the dynamic hepatic response rather than a static change, we subjected offspring to fasting stress. The exposure to MLP diet was limited specifically to the early gestational period by employing *in vitro* fertilization and embryo transfer methods. Multi-omics analyses and meta-analytical approaches were undertaken to evaluate the nutrition-sensing hepatic response system in young offspring exposed to prenatal suboptimal nutrition.

## Results

### Effects of early gestational MLP diet on the systemic response to fasting in offspring

Mouse dams were subjected to either normal protein or low-protein isocaloric diet regimes from gestational day 1 until day 10 as described (Supplementary Table S1, Fig. S1, and see Materials and methods). Early gestational MLP diet did not affect offspring body weight at any growth stage. On postnatal day 60, for each maternal diet group, F1 males were randomized into three different feeding groups: *ad libitum* fed (Ctrl.ad_lib, control pup - *ad libitum* fed; MLP.ad_lib, MLP pup - *ad libitum* fed), fasted for 24 h (Ctrl.fasted, control pup - fasted; MLP.fasted, MLP pup - fasted), and refed for 24 h following the 24-h fasting (Ctrl.refed, control pup - refed; MLP.refed, MLP pup - refed) (Supplementary Fig. S1). Body weight and organ size were not affected by the MLP diet during the fasting/refeeding cycle (Supplementary Fig. S2a–d). Plasma glucose in both maternal diet groups decreased after fasting and increased by refeeding in a similar manner (Supplementary Fig. S2e). Plasma triacylglycerol and total cholesterol levels were relatively stable during the fasting/refeeding cycle regardless of maternal diet, except that total cholesterol levels increased after fasting only in the control pups (Ctrl.fasted) (Supplementary Fig. S2f,g). In contrast, non-esterified fatty acids (NEFA) and 3-hydroxybutylate levels increased in the fasted state. Importantly, the increase in the levels of NEFA and 3-hydroxybutylate was lower in the MLP pups (Supplementary Fig. S2h,i), which resembles the phenotype commonly seen in metabolic disease-model mice^16^. Overall, the data shows that early gestational MLP diet does not change the steady-state metabolic phenotype of offspring at 8–9 weeks of age, but changes the lipid regulation in response to 24-h fasting.

### Prenatal exposure to MLP diet in early gestation significantly changed hepatic transcription in young adult mice only in the fasted state

**Figure 1:**
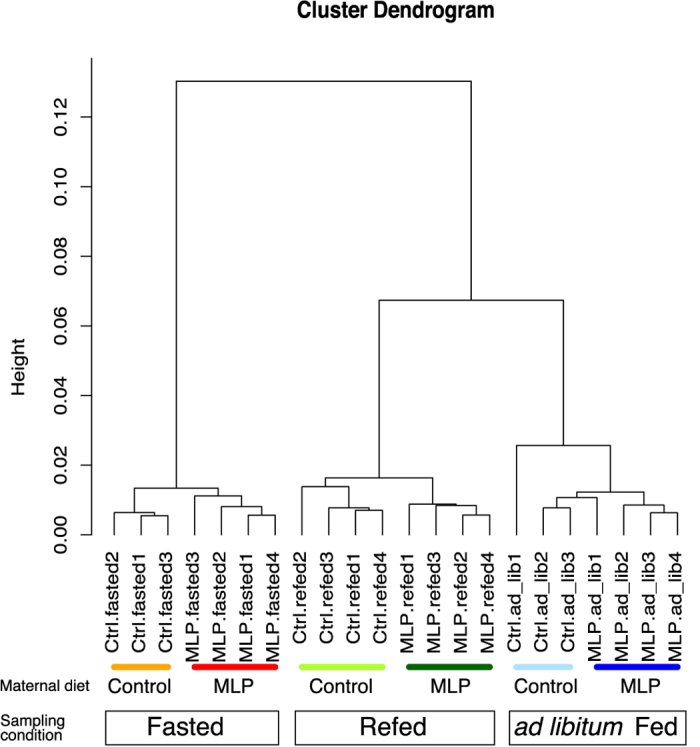
Unsupervised hierarchical clustering of microarray data. Clustering was based on normalized expression data of all samples with the 31,336 transcript-detecting probes. Spearman’s metric and Ward’s linkage methods were applied to draw the dendrogram. MLP, maternal low protein.

We examined whether early gestational MLP diet affected the hepatic transcriptional profile. Gene expression profiles of at least three randomly chosen offspring samples per group (Ctrl.ad_lib, MLP.ad_lib, Ctrl.fasted, MLP.fasted, Ctrl.refed, MLP.refed) were analyzed using microarrays as described in Materials and methods. A total of 59,305 probes covering the full set of mouse gene transcripts were represented in the array, of which 31,336 were detected in the liver. We first assessed whole transcriptional differences among offspring caused by maternal diet (control or MLP) and feeding-fasting conditions (*ad libitum* fed, fasted, or refed). Unsupervised hierarchical clustering analysis showed two distinct clusters corresponding to fed and fasted states. Although maternal diets further differentiated into two distinct clusters in fasted (Ctrl.fasted vs. MLP.fasted) and refed (Ctrl.refed vs. MLP.refed), but not in ad libitum fed (Ctrl.ad_lib vs. MLP.ad_lib) state (Fig. 1), probes detecting differentially expressed transcripts between control and MLP were identified in the fasted state only (Table 1). Significant differentially expressed genes (DEGs) at the Entrez Gene level were ranked as described in Methods. A total of 111 DEGs were identified, of which 36 and 75 genes had lower and higher expression levels, respectively, in MLP pups than in control pups (Supplementary Fig. S3a–c). Bioinformatic analyses using functional annotation analysis (DAVID) revealed a significant overrepresentation of stress response, response to unfolded protein, long-chain fatty acids metabolic process, and chaperone categories among genes downregulated in MLP pups (Supplementary Fig. S3d). qRT-PCR assays verified the significantly lower expression of Hsp70-Hsp90-Hsp40 molecular chaperone complex genes (*Hspa1a, Hspa1b, Hsp90aa1, Dnaja1*, and *Dnajb1*) in MLP pups in the fasted state. The transcriptional levels of co-chaperone or molecules interacting with Hsp70-Hsp90 complex genes (*Hsph1*, *Bag3, Stip1*, and *Hspb1*) were significantly decreased in MLP pups as well. The levels of *Acot2, Acot3*, and *Acot4* transcripts were lower in MLP pups, and a significant difference was verified in *Acot3* (Supplementary Fig. S4).

**Table 1:** Number of probes with significantly different signal intensities between control and MLP at three different nutritional conditions.

Number of probes significantly different between control and MLP (Out of 31336 probes)			
FC, WAD	|FC| > 1.5 & rank of WAD < 3000		
BH method	FDR value < 0.05	FDR value < 0.1	FDR value < 0.2
*ad libitum* fed	0	0	0
fasted for 24 h	73	218	398
refed for 24 h	0	0	222

MLP, maternal low protein**;** FC, fold-change; WAD, weighted average difference; FDR, false discovery rate; BH method, Benjamini and Hochberg’s method.

### Transcriptional response to fasting was deteriorated in MLP pups

**Figure 2:**
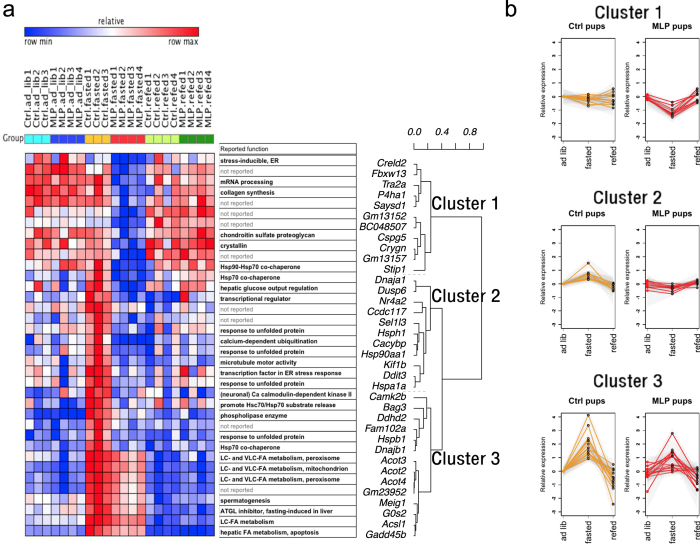
Heat map visualization of the transcriptional profile of feeding-fasting differentially expressed genes. The feeding-fasting kinetic profile of 36 DEGs (lowered in MLP at the fasted state) was analyzed. (**a**) A heat map was drawn on the GENE-E platform (https://software.broadinstitute.org/GENE-E/index.html). The subjects of the same group (ctrl.ad lib, N = 3; MLP.ad lib, N = 4; ctrl.fasted, N = 3; MLP.fasted, N = 4; ctrl.refed, N = 4; MLP.refed, N = 4) are clustered together in columns. Relative expression is depicted in red (high expression) or blue (low expression) for each gene. Genes are grouped in rows according to the similarities in expression changes by maternal diet and nutritional fluctuation as shown in the dendrogram. Reported gene functions are shown. Hierarchical clustering of pairwise Pearson’s correlation coefficients identified three clusters. (**b**) For each cluster, the mean and SD (relative to mean expression value of control *ad libitum*) was calculated with log_2_-transformed expression data per each group and shown as a graph (orange line, control; red line, MLP; gray zone, ± SD). MLP, maternal low protein; DEG, differentially expressed gene.

Because significant differences in gene expression were observed only in the fasted state, we asked whether the MLP diet mainly affected the dynamic transcriptional response to fasting rather than the constitutive basal levels. The group average of normalized expression patterns for *ad libitum* fed, fasted, and refed conditions were analyzed for the above 111 DEGs, and then a correlation coefficient matrix for either MLP low DEGs or MLP high DEGs was hierarchically clustered (Fig. 2 and Supplementary Fig. S5). Among 36 DEGs with lower expression levels in MLP pups in the fasted state, clusters 2 and 3 (corresponding to about 70% of total) corresponded to fasting-inducible genes (Fig. 2). Importantly, stress response, response to unfolded protein, long-chain fatty acids metabolic process, and chaperone genes all belonged to these clusters.

The stress response and response to unfolded protein categories included *Hspa1a/Hspa1b*, *Hsph1, Hspb1, Hsp90aa1, Bag3*, and *Dnajb1*. To confirm that these genes were upregulated after a 24-h fasting, we performed a meta-analysis of three independent transcriptomics datasets of mouse livers in equivalent conditions (*ad libitum* fed vs. 24-h fasting), age (10–16 weeks old), gender (male), and strain (C57BL/6 wild type) (SI datasets: GSE51712, GSE46495, and GSE39313). General 24-h fasting DEGs were composed of 655 upregulated and 781 downregulated genes (SI datasets 2 and 3). Among them, *Hspa1a/Hspa1b* was commonly upregulated (Supplementary Table S2). The expression levels of *Hsph1, Hspb1, Hsp90aa1, Bag3*, and *Dnajb1* were higher in the fasted state than in the fed state in all GEO datasets, but this difference was statistically significant in only one out of three datasets (Supplementary Table S2).

The long-chain fatty acid metabolic processes category includes *Acot3, Acot2, Acot4*, and *Acsl1*. These genes were induced by fasting and were significantly upregulated in at least two microarray experiments (Supplementary Table S2).

**Figure 3:**
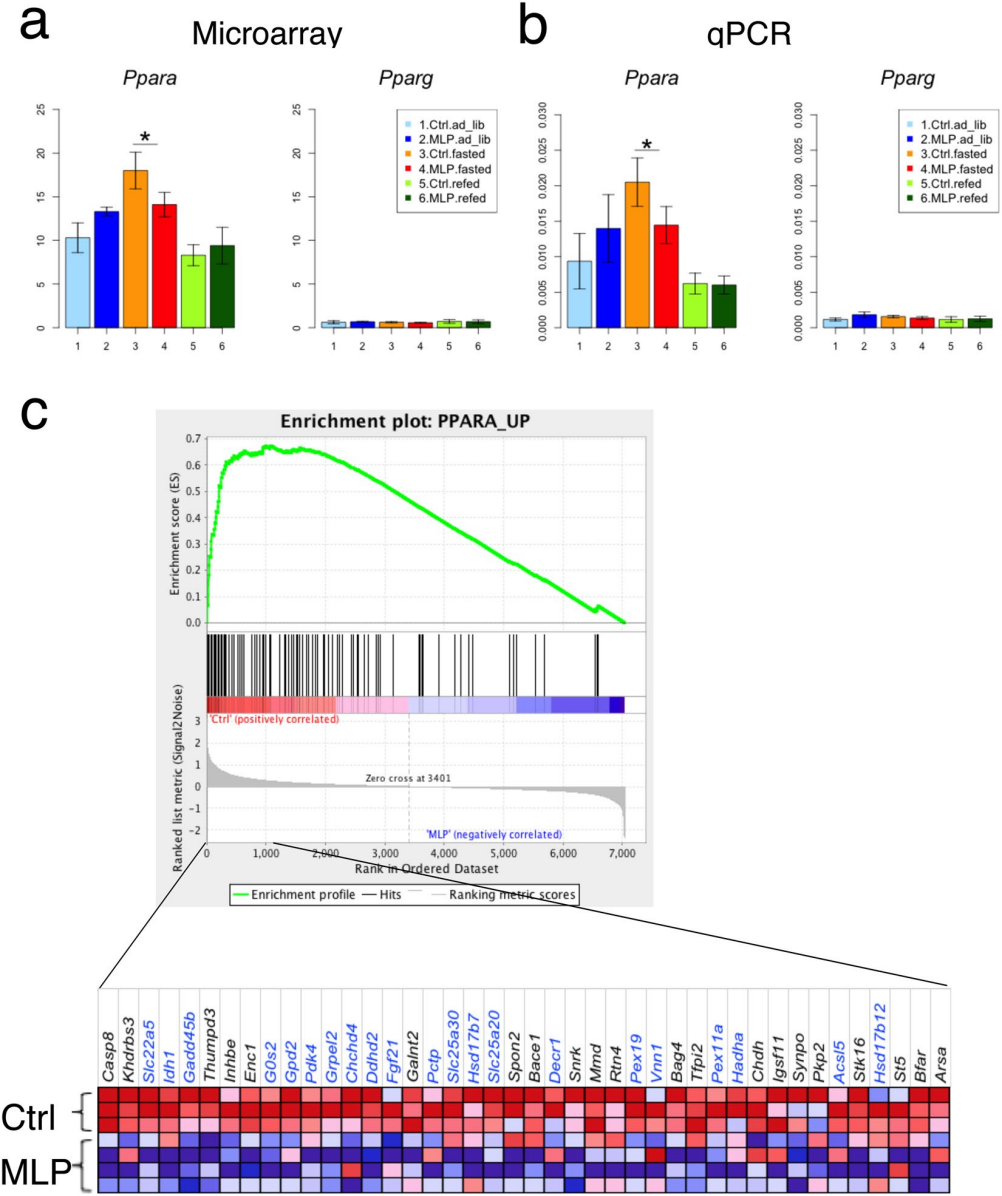
Effects of MLP diet on *Ppara* gene expression and its target genes in the fasted state Expression differences between control and MLP pups of *Ppara* and its target genes were tested. (**a**) Microarray expression data of *Ppara* and *Pparg* are shown. Values are 75%-tile normalized (N = 3–4 animals/group). (**b**) qPCR validation of *Ppara* and *Pparg* expression (N = 7 animals/group). Data are shown as mean ± SD. *P < 0.05. (**c**) PPARα inducible gene sets were prepared based on the PPARα tailored analysis described by Szalowska *et al*.^19^. The list of gene components and their biological function are shown in dataset 4. Gene Set Enrichment Analysis^20^ showing that PPARα-mediated gene upregulation was significantly decreased in MLP pups (normalized enrichment score = 1.43, FDR < 0.022). A heatmap is provided illustrating gene expression levels for each gene in the core enrichment subset (blue, low; red, high). Genes related to lipid metabolism are denoted in blue.

Acyl-CoA thioesterases not only regulate PPARα activity by controlling intracellular levels of PPARα ligands^17^ but also are themselves PPARα targets. Among the above mentioned stress response genes downregulated in the MLP pups, *Hspa1a/Hspa1b*, *Hsph1, Dnaja1*, and *Cacybp* were previously reported to be the hepatic targets regulated by PPARα, not only by HSF1^18^. Concordantly, *Ppara* upregulation in response to fasting were impaired in the MLP pups (microarray, FDR < 0.18; qPCR, p < 0.0029) (Fig. 3a,b). Among several fasting-responsive transcription factors^13^, *Ppara* was the only factor whose expression was dysregulated in the MLP pups (Supplementary Fig. S6c(6)). These results suggest that MLP diet might affect the genes induced in response to fasting and related to the PPARα pathway.

### MLP diet affected PPARα-mediated pathways

We tested whether the transcription of PPARα-inducible genes in response to fasting was affected by the MLP diet. For this purpose, we selected 224 PPARα upregulating (18 functions) genes recently identified as PPARα-tailored targets by Szalowska *et al*.^19^ (SI dataset 4), and performed Gene Set Enrichment Analysis^20^ to evaluate the differences between control and MLP pups. Figure 3c shows that a set of PPARα-upregulating genes were significantly suppressed in the MLP pups (normalized enrichment score = 1.43, FDR < 0.022), the half of which are relevant to lipid metabolism (shown in blue).

**Figure 4:**
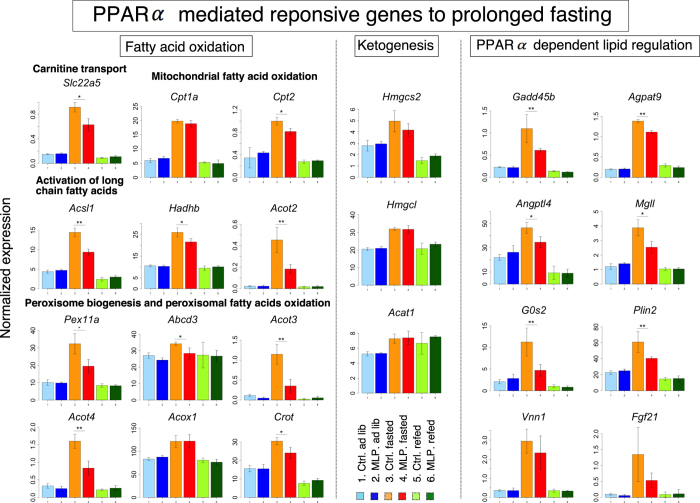
Effects of early maternal low protein diet on transcriptional response to 24-h fasting. The microarray expression data for the representative genes involved in 24-h fasting response are shown. Values were 75% tile-normalized. Data represent the mean ± SD (N = 3–4 animals/group). *P < 0.05; two-tailed Student’s t-test. **FDR < 0.1; Benjamini and Hochberg’s method.

We next studied in detail the effects of MLP diet on fatty acid oxidation, ketogenesis, and lipid metabolism, in which PPARα plays a crucial role in a prolonged (24-h) fasted state (Fig. 4). The induction of most genes involved in fatty acid oxidation was uniformly and weakly diminished in the MLP pups (Supplementary Fig. S6b (1)-(9)), although the expression of neither *Cpt1a* nor *Acox1* was affected (Fig. 4). The expression of genes involved in ketogenesis (*Hmgcs2, Hmgcl*, and *Acat1*) was not changed at all by MLP diet. In contrast, the genes involved in lipid regulation (*Gadd45b, Agpat9, Angptl4, Mgll, G0s2*, and *Plin2*) were significantly downregulated in MLP pups (Fig. 4).

Genes for gluconeogenesis regulated by the CREB pathway (*G6pc, Pck1, Tat*, and *Pcx*) were similarly induced both in control and MLP pups, whereas genes induced by PPARα (*Gpd2, Gpd1*, and *Gyk*) were not upregulated in MLP pups (Supplementary Fig. S6c, (1) and (2)). In addition, gene expression of enzymes for amino acid degradation (*Prodh* and *Ass1*) was not downregulated in the MLP pups, indicating that PPARα signaling was impaired in the MLP pups (Supplementary Fig. S6b (10)). Although PPARγ shares the same intracellular PPAR ligands with PPARα, the targets (*Cd36* and *Mogat1*) specific to PPARγ^21^ were not affected by MLP diet (Supplementary Fig. S6c (7)). Genes related to citrate cycle, glycolysis, and lipogenesis were not affected by MLP diet as shown in Supplementary Fig. S6c (3–5). The data suggest that part of PPARα signaling in the fasted state was impaired by early gestational MLP diet, although the typical PPARα target genes were minimally affected.

### Effects of early gestational MLP diet on the hepatic metabolome

**Figure 5:**
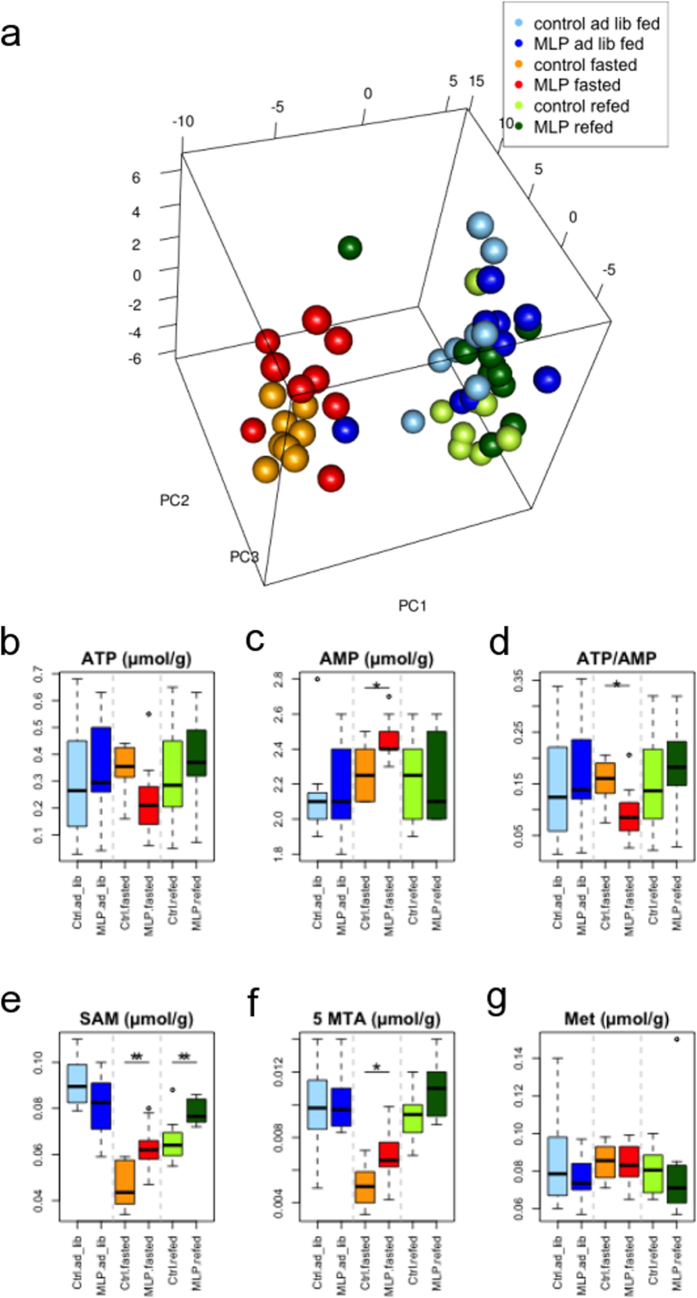
Hepatic metabolite analyses. The effect of MLP diet on hepatic metabolome was analyzed. (**a**) Score plot of a principal component analysis (PCA) based on the liver metabolite profiles of all animal samples is shown. The proportion of variance for PC1, PC2, and PC3 are 32.3%, 13.6%, and 10.4%, respectively. (**b**–**g**) Hepatic concentrations of metabolites or the ATP/AMP ratio in six animal groups (control ad lib, N = 8; MLP ad lib, N = 10; control fasted, N = 8; MLP fasted, N = 9; control refed, N = 8; MLP refed, N = 10) are shown as boxplots. Assays were conducted as described in Materials and methods. Boxes indicate the inter-quartile range of 25–75% and median. Whiskers show the minimum and maximum of all the data. *P < 0.05, **P < 0.01 for control vs. MLP; two-tailed Student’s *t*-test. SAM, *S*-adenosyl-L-methionine; 5 MTA, 5′-methylthioadenosine (a SAM catabolite); Met, L-methionine; MLP, maternal low protein.

To compare the overall metabolic profiles of all the animals, we performed principal component analysis on the 85 detectable metabolites except for lipids in primary pathways (Methods). Fed and fasted states showed two distinct clusters, although maternal diets did not further differentiate into subgroups (Fig. 5a). Twenty–six metabolites out of 85 had concentrations that were different between the control and the MLP pups in at least one of the three feeding conditions (Supplementary Table S3).

Hepatic ATP levels were stable during the fasting-feeding cycle in the control pups. In contrast, ATP levels decreased in the fasted state in the MLP pups (Fig. 5b). Of note, the hepatic AMP levels in the fasted state were greater in the MLP than in the control pups (*P* value < 0.024) (Fig. 5c). Thus, the ATP/AMP ratio in the MLP pups was significantly lower than in the control pups (*P* value < 0.021) (Fig. 5d). On the other hand, the levels of hepatic *S-*adenosylmethionine and its catabolite, 5-methylthioadenosine, in the fasted state were higher in the MLP than in the control pups (*P* values < 0.0056 and < 0.024, respectively) (Fig. 5e,f). *S*-Adenosylmethionine is synthesized from ATP and methionine. Because methionine levels were invariant regardless of nutritional fluctuation and maternal diet (Fig. 5f), these results suggest that the balance between ATP consumption and *S*-adenosylmethionine production could be dysregulated by the MLP diet. In summary, early gestational MLP diet changed the ATP/AMP ratio and the concentration of *S*-adenosylmethionine in fasted liver.

### Effects of early gestational MLP diet on the hepatic lipid profile

**Figure 6:**
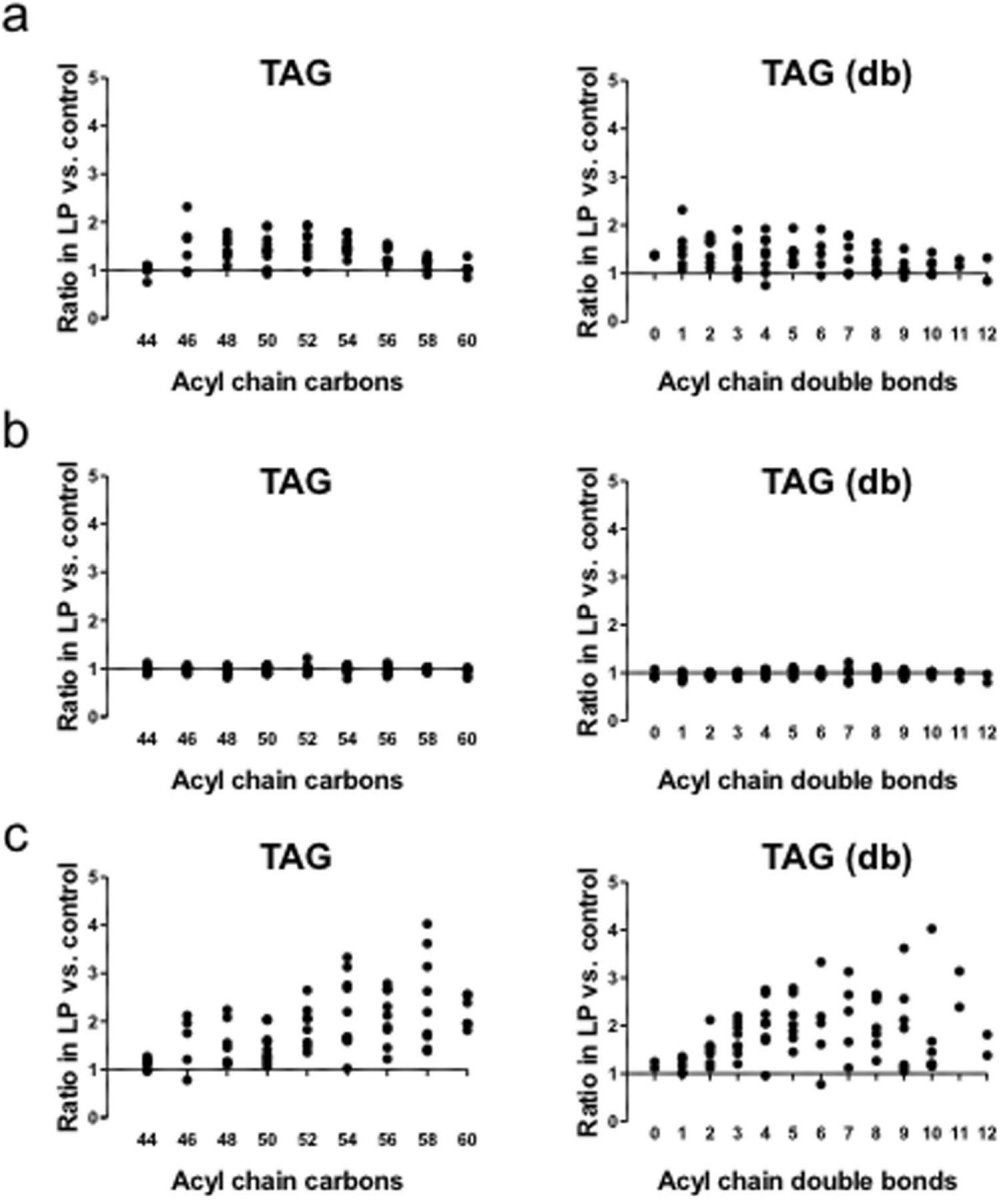
Hepatic profile of triacylglycerol acyl chain length and saturation was altered by the MLP diet. Hepatic triacylglycerol (TAG) profiles are shown according to Rhee *et al*.^15^ using the geometric mean ratio of TAG levels in MLP versus that in controls (Figure S7). Each circle represents a distinct TAG, organized along the x-axis based on total acyl chain carbon number (left) and double bond content (right), calculated from data shown in Figure S7. Data are shown for each nutritional condition: (**a**) *ad libitum* fed, (**b**) fasted, and (**c**) refed. MLP, maternal low protein.

We tested whether hepatic triacylglycerol composition was changed by early gestational maternal diet (Fig. 6 and Supplementary Fig. S7), because qualitative as well as quantitative changes in lipids are in accordance with pre-diabetic^14^ or PPARα-defective conditions^22^. In the refed state, the levels of triacylglycerols with a higher number of carbon atoms and double bonds were elevated in MLP compared to control pups, which is shown in Fig. 6c as an upsloping relation between the ratio of MLP to control and either carbon atom number or double bond content. Thus, lipid profiling analysis suggests that early gestational MLP diet affects hepatic fatty acid metabolism and leads to an altered triacylglycerol composition.

## Discussion

This study investigated the effects of early gestational MLP diet on the liver of young adult male mouse offspring. Our study has two important aspects. First, the MLP exposure was strictly restricted to the early gestational period. Second, the vulnerability of offspring to stress, such as fasting, was evaluated. We observed that early gestational MLP diet impairs fasting-induced transcriptional upregulation of stress response *(Hspa1a/Hspa1b, Hsp90aa1*, and others) and long-chain fatty acid metabolism (*Acot2/3/4, Acsl1*, and *Gadd45b*) genes (Figs 2–4 and Supplementary Fig. S3d). MLP-downregulated stress response genes were overlapped with transcriptionally PPARα-dependent genes^18^. Acyl-CoA thioesterases are responsible for the generation of intracellular PPARα ligands^17^. Concordantly, *Ppara* induction was suppressed (Fig. 3) and the PPARα-mediated pathway was partially downregulated in MLP pups. (Figs 3, 4 and Supplementary Fig. S6). We also found that hepatic energy availability in the fasted state was reduced in the MLP pups (Fig. 5). In addition, the relative abundance of hepatic triacylglycerols with longer acyl chains and higher saturation was higher in the MLP pups after a fasting/feeding cycle (Fig. 6c). These data suggest that the metabolism of long-chain fatty acids at prolonged fasting state is dysregulated in MLP pups.

Epidemiological studies have indicated that early, but not mid or late gestation, is a critical time-period for blood methylome variation induced by prenatal environment^8, 23^. The causal relation between methylation pattern and disease phenotype is not yet clear. Most methylation differences at birth are resolved during child development^24^. However, importantly, the molecular mechanisms underpinning metabolic disorders derived from prenatal perturbation may vary depending on the timing of exposure. The early gestational period, particularly, corresponds to the developmental stage of cell fate decision and differentiation. Therefore, it is important to evaluate nutritional perturbations in a restricted gestational time-period, which has not been routinely performed in animal studies. We restricted the period of nutritional disturbance strictly to early gestation by *in vitro* fertilization followed by embryo transfer (Supplementary Fig. S1). These methods enabled us to exclude various confounding effects of pre-conceptual and pregnancy variables.

Metabolic disorders such as dyslipidemia, steatosis, and type 2 diabetes are all multifactorial diseases, and prenatal environment is known to be a risk factor^25^. Early life period could therefore be the target of prophylactic intervention^25^. However, it is unclear whether an intervention would be beneficial to subjects with no apparent disorder, and evaluation of intermediate phenotypes is important in this context. Therefore, we focused on identification of hepatic transcriptomic and metabolomic changes in young offspring that were seemingly healthy and unchanged in steady (fed) state by the MLP diet. Indeed, early gestational MLP diet apparently did not change body weight, tissue size, or plasma glycolipid parameters of *ad libitum* fed offspring at 9 weeks of age (Supplementary Fig. S2), which is consistent with previous reports^6^. It is of note, however, that the level of circulating NEFA after 24-h fasting was lower in MLP pups and it resembled the phenotype commonly observed in mouse models of metabolic dysfunction (young *db/db*, young New Zealand Obese, and aged mice), which were recently found to be associated with dysregulation of hepatic *Gadd45b* induction^16^. Since there was no difference in weight decrease of white adipose tissue between control and MLP groups (Supplementary Fig. S2d), the difference in clearance rather than production of NEFA might explain the altered systemic lipid profile of the MLP group in the fasted state. Indeed, we found that hepatic *Gadd45b* induction upon fasting was impaired in MLP pups, as described below.

Gene expression microarray experiments have detected changes in hepatic transcription induced by maternal diet, but, importantly, only in the fasted state (Fig. 1 and Table 1). One hundred and eleven Entrez genes were identified as differentially expressed in the fasted state between control and MLP pups, of which 75 and 36 genes had higher and lower expression, respectively, in the MLP than in the control group (Supplementary Fig. S3). Whereas bioinformatic analyses (using DAVID, Reactome and other available tools) did not retrieve any enriched annotation category among genes with higher expression in MLP pups, they revealed a significant overrepresentation of stress response/unfolded protein binding and long-chain fatty acids metabolic process categories among genes downregulated in the MLP pups (Supplementary Fig. S3). These enriched categories are part of the hepatic signature of the fasting response^26^ (Fig. 2). However, some stress response/unfolded protein binding genes were not only upregulated after 24-h fasting, but also 2–4 h after refeeding conditions. Therefore, the universality of induction of stress response/unfolded protein binding genes by fasting was confirmed by meta-analysis of three independent microarray datasets with similar experimental settings (Supplementary Table S2).

In our study, *Hspa1a/Hspa1b* induction in response to fasting was primarily abolished by early gestational MLP diet. At the same time, fasting-dependent induction of *Hsp90aa1, Stip1*, and *Hsph1* was also impaired by MLP diet (Fig. 2 and Supplementary Fig. S3). *Hspa1a/Hspa1b* encodes the cytoplasmic Hsp70 proteostatic chaperone, which promotes cell survival and potently protects from apoptosis^27–29^. Because induction of components of the HSP90 chaperone complex was simultaneously suppressed in the MLP pups, it is possible that proteostasis of many HSP90 client proteins was affected. Of note, PPARα is a client of the HSP90 complex^30^. However, it is unlikely that HSP90 influenced the other pro-steatosis nuclear receptors such as PPARγ and glucocorticoid receptor (GR); *Pparg* expression levels were quite low in our experiments (Fig. 3), and GR acts as a *Ppara* inducer during prolonged fasting^31^. *Hspa1a/Hspa1b* are induced by various proteotoxic stress agents such as temperature, chemicals, toxins, heavy metals, and nutritional state (either adaptation to fasting stress or hypermetabolic, catabolic metabolic stress)^32^. Hepatic *Hspa1a/Hspa1b* can be upregulated after 24-h fasting (GSE46495 and GSE51712) or by PPARα agonists^33^. Moreover, *Hspa1a/Hspa1b*, *Hsph1, Dnaja1*, and *Cacybp* are common hepatic targets regulated by HSF1 and PPARα^18^, indicating that the genes affected by MLP diet might be transcriptionally dependent on PPARα. It is also known that hepatic transcription of *Hspa1a/Hspa1b, Hsp90aa1, Stip1*, and *Hsph1* is periodic^34, 35^, and this transcriptional rhythm is thought to be generated by changes in stress levels sensed by the animal’s body^34^. Thus, the impairment of chaperone gene induction in the MLP pups in our experiments might be caused by both decreased PPARα activity and inactivation of systemic signaling.

Early gestational MLP diet also diminished induction of another gene set, *Acot2, Acot3, Acot4*, and *Acsl1*, which are all PPARα targets (Fig. 2). Because ACOT genes are involved in the production of intracellular endogenous PPARα ligands^17^, these ligands may be dysregulated. Furthermore, the induction of *Ppara* was diminished in MLP pups and the induction of PPARα pathway genes was downregulated (Figs. 3,4 and Supplementary Fig. S6). Although the typical PPARα targets (*Cpt1a* and *Acox1*) were not affected, expression of genes involved in triacylglycerol turnover (*Mgll* and *G0s2*), regulation of hepatic and systemic lipid metabolism (*Gadd45b*), and phospholipid metabolism (*Ddhd2*) were significantly reduced (Fig. 4 and Supplementary Fig. S6). Especially, the impairment of *Gadd45b* induction in MLP pups was of special interest. It was recently uncovered that acute hepatic induction of *Gadd45b* is vital to protect liver under fasting stress by preventing accumulation of hepatic long-chain fatty acids^16^. Furthermore, the impaired GADD45b expression correlates well with metabolic dysfunction^16^. In our MLP pups, downregulation of *Acsl1* and *Gadd45b* might lead to the reduction of activated long-chain fatty acids to be further metabolized. Consistently, the low hepatic ATP/AMP ratio was observed in the MLP group (Fig. 5). Interestingly, in addition, S-adenosylmethionine production was not strongly suppressed in the fasted state in MLP pups, regardless of the invariance in methionine levels (Fig. 5). Therefore, the decline in hepatic ATP might be also partly reinforced by the consumption of ATP by virtue of S-adenosylmethionine production. After a fasting and refeeding cycle, the content of triacylglycerols with a higher number of carbon atoms and double bonds increased in MLP compared to control pups. The expression of genes involved in fatty acid elongation and desaturation was not altered in MLP pups (Fig. 6c (5)). We speculate that the change in triacylglycerol profile in the MLP group might reflect the reduction of hepatic long-chain fatty acids metabolism due to insufficient induction of *Acsl1*, ACOT, and *Gadd45b* genes in response to fasting.

We acknowledge that there are some limitations in our study. First, the transcriptional aberration in MLP pups was identified in PPARα activity-dependent genes involved in long-chain fatty acid metabolism in response to fasting. It should be determined by further work whether this transcriptional alteration results in the altered levels of the corresponding proteins. Nevertheless, our metabolome analysis results showing the decreased ATP/AMP ratio and the altered lipid profile are consistent with an impairment of long-chain fatty acid metabolism in MLP pups. Second, the loss of gene induction observed in our experiments may not be directly linked to the causes of future hepatic steatosis. However, reduced PPARα-mediated hepatic activity is correlated with development of this disease^22, 36^. Particularly, the reduced *Gadd45b* expression in fasting found in MLP pups might be related to the development of metabolic disorders. In addition, impaired proteostasis generally causes age-associated pathological conditions. Because the liver in the MLP group showed a lower expression of chaperone genes, we speculate that proteostasis activity may also be lowered in the MLP group. Third, MLP diet amplifies age-related epigenetic changes^11^, but it is unclear how ageing is involved. The epigenetic regulation of hepatic chromatin is largely driven by transcription factors responsive to nutritional fluctuation. If the fasting response is changed by MLP diet, the epigenetic differences produced after each fasting will be amplified over time. Our study demonstrated that early gestational MLP diet affects the transcriptional and metabolic response to fasting. Future studies will be required to examine whether repeated nutritional challenges can result in epigenetic differences between control and MLP groups. Forth, our experiments were performed in three nutritional conditions only: *ad libitum* fed, fasted for 24 h, and refed for 24 h following the 24-h fasting. Because the response to fasting of most genes involved in hepatic metabolism peaks after 24 h of starvation^26^, transcriptomic analysis in this study was performed to clarify the effects on the fasting response. However, proteostatic chaperones also play an important role in the hypermetabolic, catabolic response to metabolic stress. *Hspa1a/Hspa1b*, *Hsp90aa1*, *Hpsh1*, and other chaperones are also transiently induced after refeeding following starvation. The induction peaks of those genes are different from each other. Future work with time series experiments is required to clarify the effects of the MLP diet on the metabolic stress response. Lastly, this study focused on identification of early hepatic transcriptomic and metabolomic signs of dysregulated metabolic response. The mechanisms whereby the prenatal environment impairs such dynamic response were not investigated. Further biochemical and molecular studies are required to confirm our results. Specifically, it might be important to clarify whether AMPK and the relevant molecules are involved in the partial impairment of the PPARα pathway caused by MLP diet.

In summary, multi-omics analyses revealed that early gestational MLP diet alters the hepatic response to fasting even in young offspring prior to showing overt metabolic disorders. The transcriptional induction of genes of proteostatic chaperones and acyl-CoA metabolic enzymes as well as a newly identified metabolic regulator, *Gadd45b*, was impaired. Reflecting the reduced metabolic activity of long-chain fatty acids, the hepatic triacylglycerol profile of acyl chain length and saturation was changed in MLP pups. Our study provides evidence that early prenatal perturbations change hepatic gene expression and lipid metabolism by altering responsiveness to fasting. Future work will be required to assess how these early changes are linked to the development of disease.

## Methods

### Ethical statement

The study was conducted in accordance with our institution’s ethical guidelines for animal experiments. The protocols for animal handling and treatment were reviewed and approved (approval ID: S-25025) by the Animal Care and Use Committee at Tokyo Medical University.

### Animals

Animals were kept under standard temperature- and humidity-controlled conditions on a 12:12 h light (08:00–20:00)-dark (20:00–08:00) cycle. Two-cell embryos of C57BL/6 J mice obtained by *in vitro* fertilization were transferred into pseudo-pregnant ICR female mice (Sankyo Labo Service, Tokyo, Japan). The surrogate mothers (F0 dams) were randomized into two different diet groups: normal protein diet (Ctrl, 18% casein, Oriental Yeast, Tokyo, Japan) throughout the entire gestation and lactation period or low protein diet (MLP, 9% casein, Oriental Yeast) for the initial 10 days followed by normal protein diet for the rest of the gestation and lactation period. At delivery, litters were standardized to ~8 offspring. The offspring were weaned onto a standard laboratory chow diet on postnatal day 20. On postnatal day 60, F1 males were randomized into three different feeding groups: *ad libitum* feeding, 24-h fasting, and 24-h fasting followed by 24-h re-feeding. Eight to 10 animals from each group were weighted and sacrificed. Whole blood was collected by heart puncture into ethylenediaminetetraacetic acid (EDTA)-containing tubes. Plasma was separated by centrifugation and stored at −80 °C until used in the analyses. Liver and perigonadal- and perirenal- white adipose tissues were weighted, dissected, quickly frozen in liquid nitrogen, and stored at −80 °C until used in the analyses. To negate time differences between the six groups, all samples were collected between 9:30 AM and 1:00 PM and every control and MLP sample were collected alternately. The full diet composition is shown in Supplementary Table S1 and the experimental design is shown in Supplementary Fig. S1.

### Measurement of plasma parameters

Plasma glucose, total cholesterol, triacylglycerols, and NEFA were measured by Nagahama Life Science Laboratory (Shiga, Japan).

### RNA preparation and microarray experiment

Total RNA was extracted from the left medial lobe of the liver using the RNeasy mini kit (Qiagen). RNA quality and quantity was evaluated using a NanoDrop spectrophotometer (Thermo Fisher Scientific, Wilmington, USA) and an Agilent Bioanalyzer (Agilent Technologies, CA, USA). Three animal samples from each group were randomly selected. Each RNA sample (150 ng) was used to synthesize, amplify, and label cRNA using the Low Input QuickAmp Labeling kit (Agilent Technologies). Hybridization on SurePrint G3 Mouse GE 8 × 60 K microarrays (Agilent Technologies) and scanning were performed according to the manufacturer’s instructions. All arrays met the Agilent QA/QC standards. Raw microarray data were extracted using Agilent Feature Extraction Software (ver. 11.0.1.1). Microarray data are available in the NCBI Gene Expression Omnibus under the accession number GSE100313.

### Statistical analysis of microarray data

Microarray data were 75% tile-normalized and log_2_-transformed according to the manufacturer’s recommendation^37^ using R software (ver. 3.3.1). Control features and non-uniform or below-background features were removed. Hierarchical clustering analysis with Ward’s clustering algorithm was performed using Spearman’s correlation coefficients for pairs of whole data in R. Differential expression in features was determined using the empirical Bayes statistics for differential expression (eBayes) framework^38^ (*limma* ver. 3.30.4). The moderated t-statistic *P* values derived from *limma* were further adjusted for multiple testing by Benjamini and Hochberg’s method to control for false discovery rate (FDR). To reduce the inclusion of weaker signals falsely detected as differentially expressed, the weighted average difference (WAD) method^39^ was further applied to selected DEGs. Differential expression was defined as fold-change > 1.5 or < 0.67, WAD rank < 3,000, and FDR < 0.1. When there were multiple features for the same sequence as technical replicates, the feature with the top rank of shrinkage t statistics rank^40^ value was kept as representative. Among differentially expressed features, those with Entrez ID annotation were selected as DEGs. Functional annotation, enrichment analysis, and pathway analysis of DEGs were performed using the Database for Annotation, Visualization, and Integrated Discovery (DAVID ver. 6.8)^41^.

### Validation of gene expression by quantitative real time RT-PCR

Real-time RT-PCR was carried out essentially as described^42^. All RNA samples (including 3–4 samples used for the array and an additional 4–6 samples) were analyzed. After treatment with DNase I (Invitrogen), cDNA was synthesized with Superscript III First-strand Synthesis System for RT-PCR (Invitrogen). All reactions were run together with RT minus controls. Real-time PCR was performed using the LightCycler 480 Probe Master kit, specific primers, and the corresponding Universal Probe Library probes according to the manufacturer’s instructions (Roche Applied Science). The Universal Probe Library probe IDs and primer sequences are listed in Table S6. *Rpl13a* was used as a reference gene.

### Preparation for metabolite measurements

Frozen tissue (approximately 50 mg) was plunged into methanol (0.5 mL) containing internal standards and 20 M each of methionine sulfone, D-camphor-10-sulfonic acid, and 2-(n-morpholino)ethanesulfonic acid, and homogenized at 1,500 rpm for 15 min using a Shake Master Neo (BMS, Tokyo, Japan) to inactivate the enzymes. The homogenate was analyzed by capillary electrophoresis-mass spectrometry (CE-MS) and liquid-chromatography MS (LC/MS) for profiling of charged and other metabolites, respectively.

For CE-MS analyses, 500 μL of chloroform and 200 μL of Milli-Q water were added to 470 μL of the homogenized solution, and the mixed solution was centrifuged at 4,600 × *g* for 15 min at 4 °C. The upper aqueous layer (300 μL) was centrifugally filtered at 9,100 × *g* for 3.5 h at 4 °C through a 5-kDa cutoff filter (Millipore, MA) to remove large molecules. The 150 μL filtrate was lyophilized and dissolved in 25 μL of Milli-Q water containing a reference compound (200 μM of 3-aminopyrrolidine and trimesate) prior to CE-MS analysis.

For LC/MS analysis, the homogenate of 30 µL was transferred to new tube and mixed with 50 µL of 2-propanol containing internal standards (4 µ M dodecanoyl sphingomyeline[SM(d18:1/12:0)], 1,2-dimyristoyl-sn-glycero-3-phosphatidylcholine[PC(14:0/14:0)], and 40 µM etodolac). The mixture was divided in two aliquots to analyze fatty acid and acyl carnitine or triacylglycerol and phospholipids. An aliquot of the homogenate (20 µL) was centrifuged at 20,400 × *g* for 10 min at 4 °C. A portion of the supernatant was injected into LC/MS for analysis of acylcarnitine and fatty acid. An aliquot of the homogenate (60 µL) was combined with 60 µL of chloroform. The mixture was left for 15 min at room temperature (20–25 °C) and then 108 µL of MeOH/water (60:40, v/v) was added. After being left at room temperature for another 15 min, the mixture was centrifuged at 20,400 × *g* for 10 min at 4 °C. A portion of the supernatant was injected into LC/MS for analysis of triacylglycerol and phospholipids.

### Metabolite measurement conditions

The instrumentation and measurement conditions used for CE-time-of-light-MS were described elsewhere^43^. The LC-MS analysis is described below. The LC system was the Agilent 1290 Infinity HPLC (Agilent Technologies, Palo Alto, CA). This system is equipped with automatic degasser, quaternary pump, and autosampler. Chromatographic separation was performed using an Acquity UPLC HSS T3 C18 column (2.1 i.d. × 50 mm, 1.7 µm; Waters, Milford, MA), and the column temperature was maintained at 45 °C. The mobile phase consisted of acetonitrile-methanol-water (3:1:1), with 5 mM ammonium formate as eluent A and isopropanol with 5 mM ammonium formate as eluent B. The initial mobile phase was 100% eluent A at a flow rate of 0.3 mL/min. The gradient profiles of eluent B were 40%, 64%, 64%, 82.5%, 85%, and 95% acetonitrile at 5, 7.5, 12, 12.5, 19, and 20 min, respectively. The injection volume was 1 µL. MS data were acquired from a 6530 Accurate-Mass Q-TOF LC/MS using the dual spray ESI of G-3251A (Agilent). Samples were analyzed by both positive and negative ion electrospray MS. The MS conditions were as follows: gas temperature 350 °C, drying gas 12 L/min, nebulizer 30 psig, fragmentor 180 V, skimmer 90 V, OCT1 RF Vpp 230 V, and scan range m/z 100–1,600. For negative and positive modes, the capillary voltages were 3.5 and 4.0 kV, respectively. Methanol (LC/MS grade), acetonitoryl (LC/MS grade), and 2-propanol (HPLC grade) were purchased form Wako Pure Chemical (Osaka, Japan). Etodolac (1,8-diethyl-1,3,4,9-tetrahydropyrano[3,4-b]indole-1-acetic acid) was purchased from Sigma-Aldrich (St Louis, MO). Dodecanoyl sphingomyeline [SM (d18:1/12:0)], 1,2-dimyristoyl-sn-glycero-3-phosphatidylcholine [PC(14:0/14:0)] was obtained from Avanti Polar Lipids (Alabaster, AL).

### Statistical analysis of metabolome data

Raw data were processed by the in-house software MasterHands^44^. The analyses started from data conversion, background subtraction, and detection of all possible peaks showing signal-to-noise ratio (S/N) > 3. Retention time correction was performed using commonly observed peaks among multiple datasets and eliminating noise and redundant features, e.g., isotopic peaks. Among remaining peaks, we identified the metabolites by matching *m/z* and corrected relation times with those of our standard libraries. Fatty acids were estimated by comparing corrected retention time and *m/z* values calculated from molecular structures. Phospholipid and triglyceride (TAG) were estimated as previously described^45^. Briefly, the phospholipid or TAG candidates were evaluated by *m/z* and the presence of fatty acid-derived peaks was confirmed in fragmented mass spectra by comparison with data in MassBank^46^.

To perform principal component analysis (*prcomp* function implemented in R), metabolite concentrations were Box-Cox-transformed and scaled to a mean of 0 and a standard deviation of 1. To identify the significant differences in concentration between control and MLP groups, the data were analyzed using Student’s *t*-test; differences with a *P* value < 0.05 were considered significant.

## Electronic supplementary material

Supplementary Information

Supplementary Dataset
