# Supplementary material for: Early gestational maternal low-protein diet diminishes hepatic response to fasting in young adult male mice
Source: Sci Rep. 2017 Aug 29;7:9812. doi: 10.1038/s41598-017-10380-4 (PMC5575317; doi:10.1038/s41598-017-10380-4)
Supplement: Supplementary file 1 — Supplementary Information [file 41598_2017_10380_MOESM1_ESM.pdf]

Supplementary Information

Early gestational maternal low-protein diet diminishes hepatic response to fasting in young adult male mice

Noriko Sato, Katsuko Sudo, Masayo Mori, Chihiro Imai, Masaaki Muramatsu, and Masahiro Sugimoto

- **Supplementary tables (Table S1-S3)**
- **Supplementary figures (Figure S1-S7)**
- **Dataset titles (1-4)**

Table S1. Experimental diet composition for the early gestational period of dams

	control diet	*LP diet
Casein	18	9
Cornstarch	35	41
Alfarized cornstarch	23	23
Sucrose	10	13
Mineral mix (AIM-93M)	3.5	3.5
Vitamin mix (AIM-93)	1	1
DL-Methionine	0.5	0.5
Cellulose	5	5
Corn oil	4	4
Total (% weight)	100	100
Total energy (kcal / 100 g)	355.6	355.7

*9 % casein low-protein diet was prepared as described by Langley and Jackson(40). Differences in the diets corresponded to not only protein content but also sucrose, cornstarch, and protein/carbohydrate ratio; however, the diet is referred to as low-protein (LP) following convention.

Table S2. The effects of 24-hr fasting on gene expression levels in three independent experimental settings

*Gene name	GSE39313		GSE46495		GSE51712	
	$Mean difference	#FDR	$Mean difference	#FDR	$Mean difference	#FDR
Nr4a2	0.03	9.41E-01	-0.03	8.90E-01	0.26	7.26E-01
Ccdc117	0.25	3.57E-01	0.45	**3.06E-02**	0.33	1.67E-01
Sel1l3	-0.22	2.97E-01	-0.26	5.64E-02	0.17	8.28E-01
Hsph1	0.97	**2.84E-05**	0.41	2.25E-01	0.67	3.37E-01
Cacybp	0.16	4.46E-01	-0.05	8.33E-01	-0.06	8.30E-01
Hsp90aa1	0.39	**2.66E-02**	0.23	3.14E-01	0.51	1.72E-01
Kif1b	0.34	**1.39E-02**	0.27	**2.99E-02**	0.34	1.29E-01
Ddit3	0.15	4.95E-01	NA	NA	NA	NA
Hspa1a	0.19	2.93E-01	0.68	**5.83E-02**	1.44	**7.47E-02**
Dnaja1	0.11	6.51E-01	0.33	2.47E-01	0.17	6.99E-01
Dusp6	-0.80	9.86E-05	-0.08	8.17E-01	-0.37	3.47E-01
Hspb1	0.40	**3.54E-02**	0.17	3.52E-01	0.63	6.21E-01
Dnajb1	0.34	**1.81E-02**	0.37	1.05E-01	0.26	4.87E-01
Fam102a	0.18	6.26E-01	0.18	1.85E-01	0.24	5.64E-01
Ddhd2	NA	NA	0.50	**2.38E-03**	0.73	1.36E-01
Bag3	0.30	1.09E-01	0.57	**6.64E-03**	0.50	1.40E-01
Camk2b	0.46	**5.06E-02**	1.31	**3.65E-03**	0.59	4.31E-01
Acot3	2.44	**2.70E-06**	1.53	**5.97E-03**	2.63	**7.85E-02**
Acot2	0.62	**5.62E-04**	1.29	**2.12E-03**	2.25	1.14E-01
Acot4	1.16	**5.75E-06**	1.11	**1.48E-03**	1.41	1.43E-01
Gm23952	NA	NA	NA	NA	NA	NA
Meig1	NA	NA	0.08	7.93E-01	0.58	1.25E-01
Acsl1	0.74	**7.21E-04**	0.24	**2.35E-02**	0.48	**9.34E-02**
G0s2	0.53	2.31E-01	0.86	2.46E-01	0.63	6.21E-01

*Of the 35 MLP downregulated genes, 24 corresponded to fasting-inducible genes. $Mean difference, log2-converted mean values in the fasted state subtracted from those in the ad libitum fed state. #FDR, q value by Benjamini and Hochberg's method. NA, not

available.

Table S3. Hepatic metabolites with different levels between control and MLP groups in each nutritional condition

Condition	KEGG	Name	Mean (S.D.) (μmol/g)		p value
			Control	MLP	
ad libitum fed	C00127	Glutathione disulfide	1.4 (0.070)	1.3 (0.10)	2.00E-03
ad libitum fed	C00148	Proline	0.34 (0.060)	0.27 (0.028)	3.91E-03
ad libitum fed	C00147	Adenine	0.011 (0.0017)	0.0095 (0.0010)	8.88E-03
ad libitum fed	C00097	Cysteine	0.014 (0.0047)	0.0090 (0.0025)	1.67E-02
ad libitum fed	C04501	*N*-Acetylglucosamine 1-phosphate	0.015 (0.0032)	0.012 (0.0019)	1.93E-02
ad libitum fed	C02918	1-Methylnicotinamide	0.0080 (0.0023)	0.0060 (0.0016)	4.01E-02
fasted	C00345	6-Phosphogluconate	0.015 (0.0030)	0.022 (0.0051)	4.39E-03
fasted	C03172	*S*-Methylmethionine	0.0052 (0.0011)	0.0080 (0.0022)	5.31E-03
fasted	C00019	*S*-Adenosylmethionine	0.046 (0.010)	0.063 (0.011)	5.67E-03
fasted	C01015	Hydroxyproline	0.049 (0.0076)	0.035 (0.010)	6.15E-03
fasted	C00015	UDP	0.044 (0.0075)	0.031 (0.0096)	7.78E-03
fasted	C00130	IMP	0.14 (0.036)	0.19 (0.033)	9.14E-03
fasted	C00114	Choline	0.45 (0.069)	0.35 (0.071)	1.08E-02
fasted	C05382	Sedoheptulose 7-phosphate	0.028 (0.0062)	0.040 (0.012)	1.39E-02

fasted	C00588	Phosphorylcholine	0.52 (0.11)	0.36 (0.12)	1.39E-02
fasted	C00055	CMP	0.024 (0.0044)	0.030 (0.0046)	1.74E-02
fasted	C00020	AMP	2.3 (0.17)	2.4 (0.12)	1.94E-02
fasted	C00170	5-Methylthioadenosine	0.0050 (0.0013)	0.0071 (0.0019)	2.39E-02
fasted	C00105	UMP	0.54 (0.094)	0.62 (0.036)	2.94E-02
fasted	C02918	1-Methylnicotinamide	0.0058 (0.0018)	0.0084 (0.0028)	4.16E-02
fasted	C00519	Hypotaurine	0.70 (0.28)	0.46 (0.17)	4.29E-02
fasted	C00135	Histidine	0.44 (0.052)	0.51 (0.075)	4.90E-02
refed	C00315	Spermidine	0.035 (0.0046)	0.028 (0.0034)	2.07E-03
refed	C00153	Nicotinamide	0.85 (0.11)	1.0 (0.11)	5.28E-03
refed	C00097	Cysteine	0.0097 (0.0026)	0.014 (0.0029)	6.65E-03
refed	C00019	*S*-Adenosylmethionine	0.066 (0.011)	0.078 (0.0054)	6.91E-03
refed	C00689	Trehalose 6-phosphate	0.037 (0.0063)	0.030 (0.0054)	3.52E-02
refed	C00065	Serine	0.33 (0.079)	0.40 (0.069)	4.89E-02

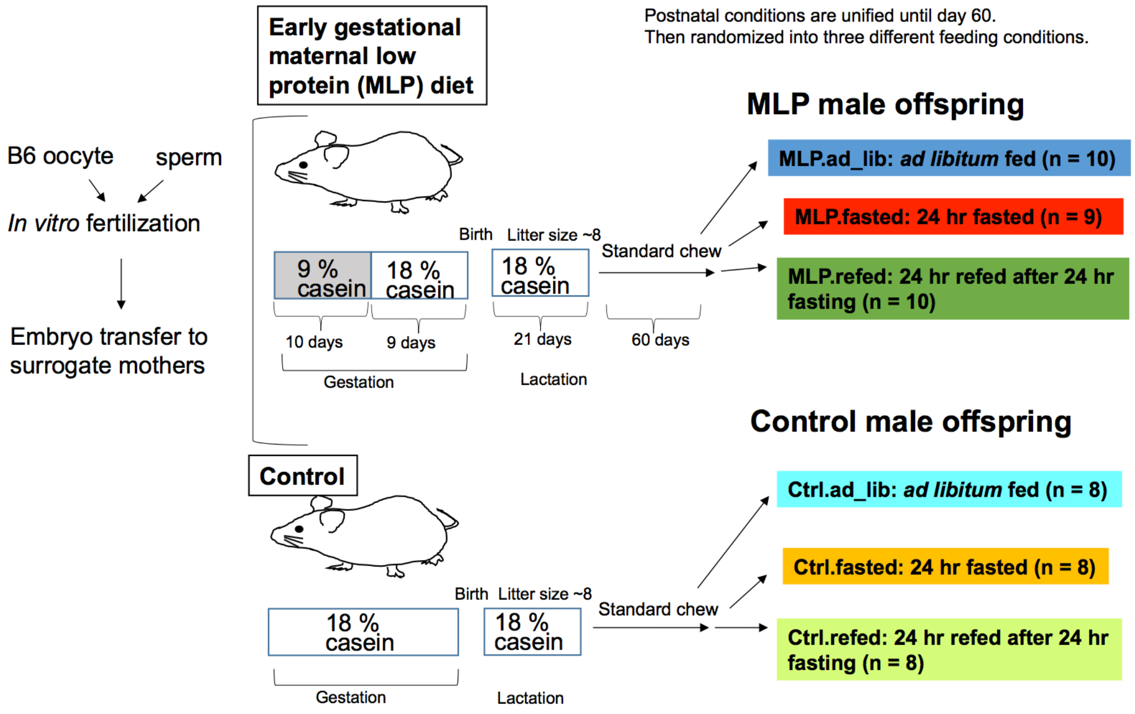

Supplementary Figure S1. **The study design.**

The timeline of the study is shown. MLP, maternal low protein.

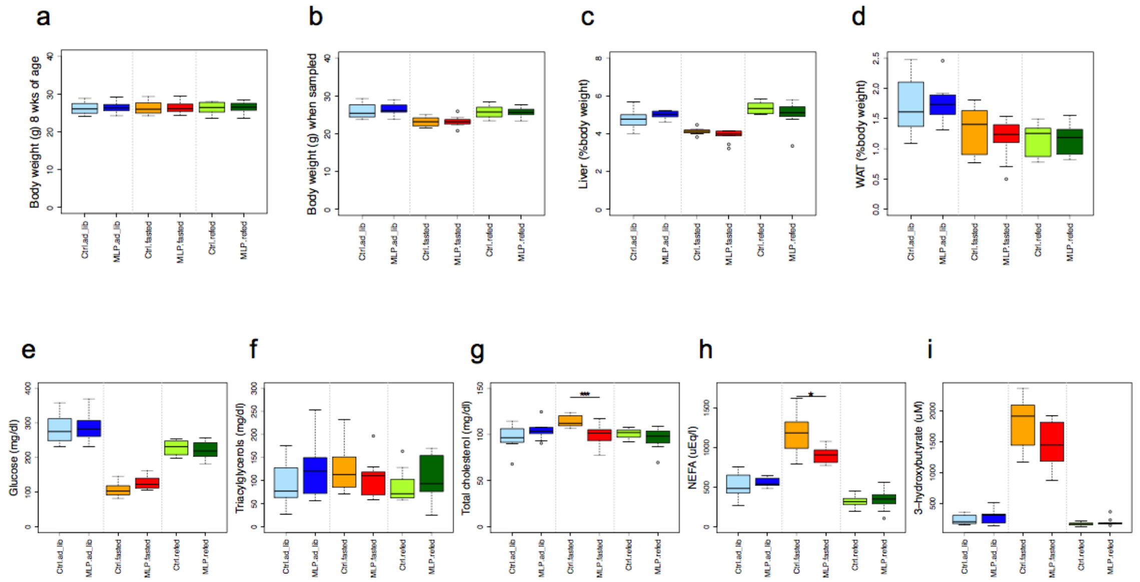

Supplementary Figure S2. **Body weight, organ size, and plasma parameters**.

The effects of different gestational maternal diets on body weight, organ size, and plasma parameters in mice at three nutritional conditions are shown. Data are presented as boxplots of (a) body weight at 8 weeks of age, (b) body weight at sampling, (c) ratio of liver weight to body weight, (d) ratio of epididymal and perirenal white adipose tissue weight (WAT) to body weight, (e) plasma glucose, (f) triacylglycerols, (g) total cholesterol, (h) non-esterified fatty acids (NEFA) and (i) 3-hydroxybutyrate. Boxes indicate the inter-quartile range of 25–75 %, and median and whiskers show the minimum and maximum of all the data. Only plasma levels of total cholesterol after fasting were influenced by maternal diet (*P <0.05, ***P <0.005; two-tailed Student's t-test). Control ad lib, N = 8; MLP ad lib, N = 10; control fasted, N = 8; MLP fasted, N = 9; control refed, N = 8; MLP refed, N = 10. MLP, maternal low protein.

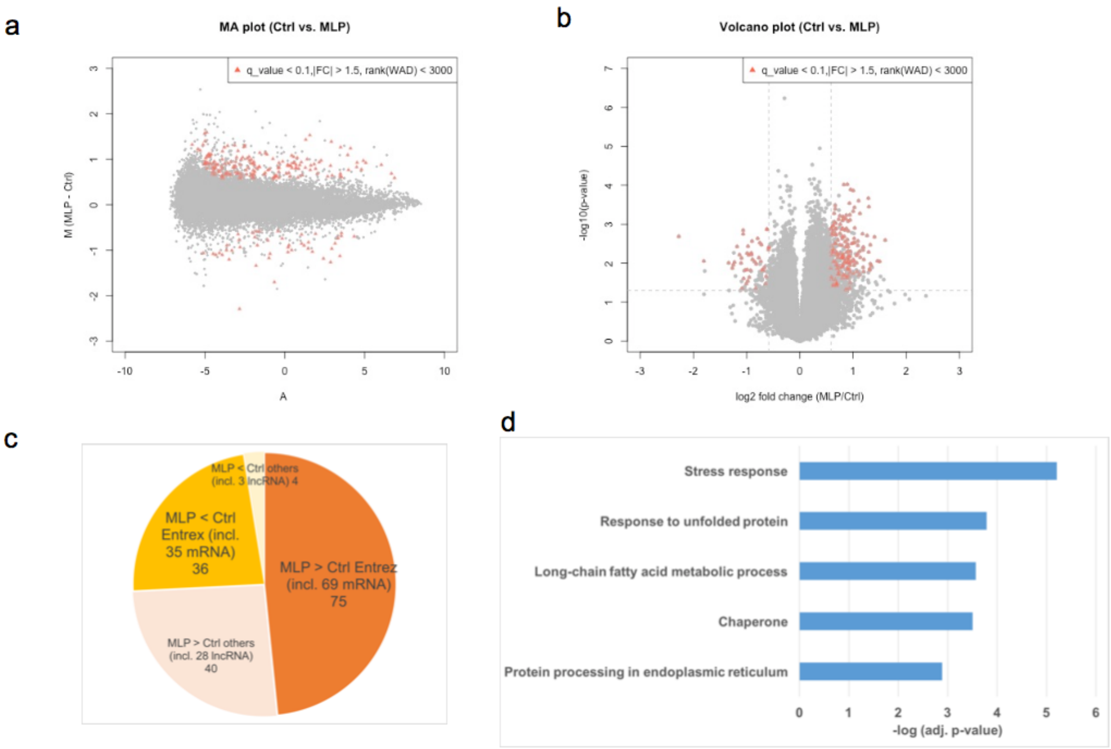

Figure S3. Differentially expressed genes between control and MLP pups in the fasted state. Differentially expressed genes between control and MLP groups were identified and functional annotation analysis was performed. (a) The x-axis represents the $\log_2$-transformed average expression and the y-axis represents the $\log_2$-transformed magnitude of changes in the MA plot. (b) The x-axis represents the $\log_2$-transformed fold-change (FC) and the y-axis represents the P value ($-\log_{10}$) in the volcano plot. Red marks represent the array features with significant differential expression ($q < 0.1$, $|FC| > 1.5$, rank of weighted average difference (WAD) statistics < 3,000) by maternal diet in (a) and (b). (c) The breakdown of the differentially expressed genes is shown. From 218 differentially expressed probes, 154 genes were uniquely identified in total. One hundred and ten out of 154 genes were annotated with Entrez ID by the manufacturer. Most of the remaining genes were long non-coding RNAs, within which *Carmn* (cardiac mesoderm enhancer-associated non-coding RNA) and *Malat1* (metastasis associated lung adenocarcinoma transcript 1) were identified as having a higher expression in MLP than the control. (d) Top 5 enriched terms of genes in functional annotation analysis. Benjamini-corrected adjusted P-values are shown. MLP, maternal low protein.

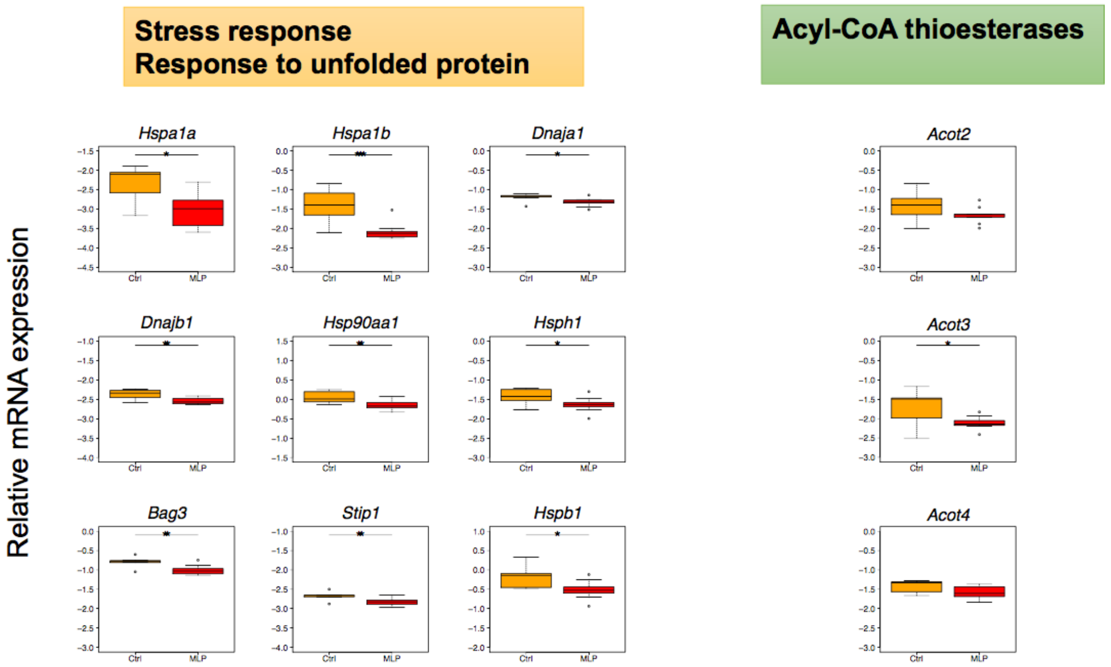

Supplementary Figure S4. **Validation of microarray results by quantitative real-time RT-PCR (qPCR) for selected genes.**

qRT-PCR validation of MLP low expression genes is shown. Values are normalized to endogenous Rpl13a expression and log10-transformed. Boxes indicate the inter-quartile range of 25–75 %, and median of data for n = 7 and n = 9 for the control and MLP groups, respectively. Whiskers show the minimum and maximum of all the data. *P < 0.05, **P < 0.01, ***P < 0.005; two-tailed Student's t-test. MLP, maternal low protein; Rpl13a, ribosomal protein L13A.

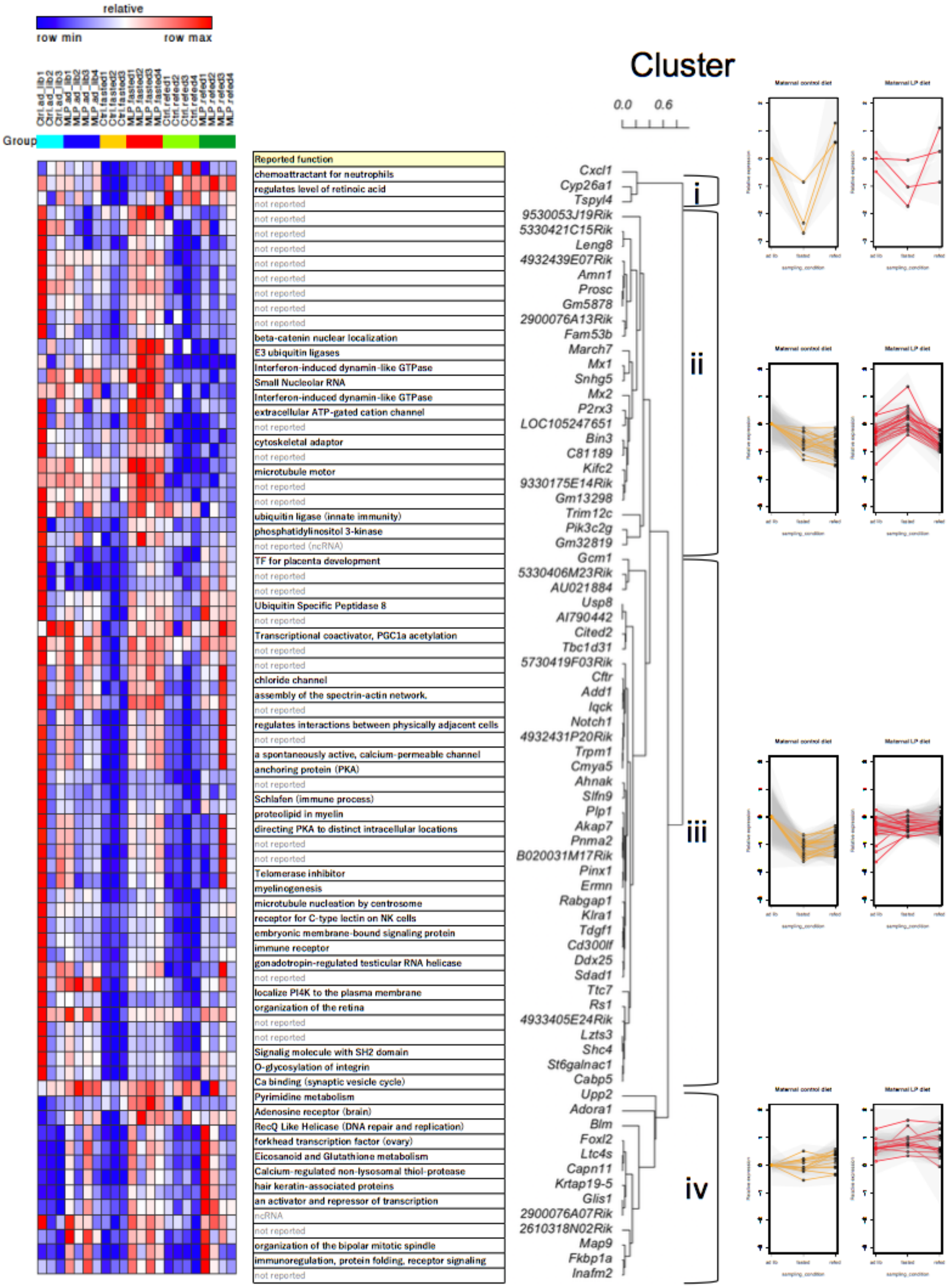

Supplementary Figure S5. Heat map visualization of expression profile of genes more highly expressed in MLP in the fasted state.

The feeding-fasting kinetic profiles of 75 DEGs (higher in MLP in the fasted state) were analyzed. A heat map was drawn on the GENE-E platform

(https://software.broadinstitute.org/GENE-E/index.html). The subjects of the same group (ctrl.ad lib, N = 3; MLP.ad lib, N =4; ctrl.fasted, N = 3; MLP.fasted, N = 4; ctrl.refed, N = 4; MLP.refed, N =4) are clustered together in columns. Relative expression is depicted in red (high expression) or blue (low expression) for each gene. Genes are grouped in rows according to the similarities in expression changes by maternal diet and nutritional fluctuation as shown in the dendrogram. Hierarchical clustering of pairwise Pearson's correlation coefficients identified three clusters.

a. Scheme summarizing the changes related to lipid metabolism observed in MLP pups at 24-h fasting

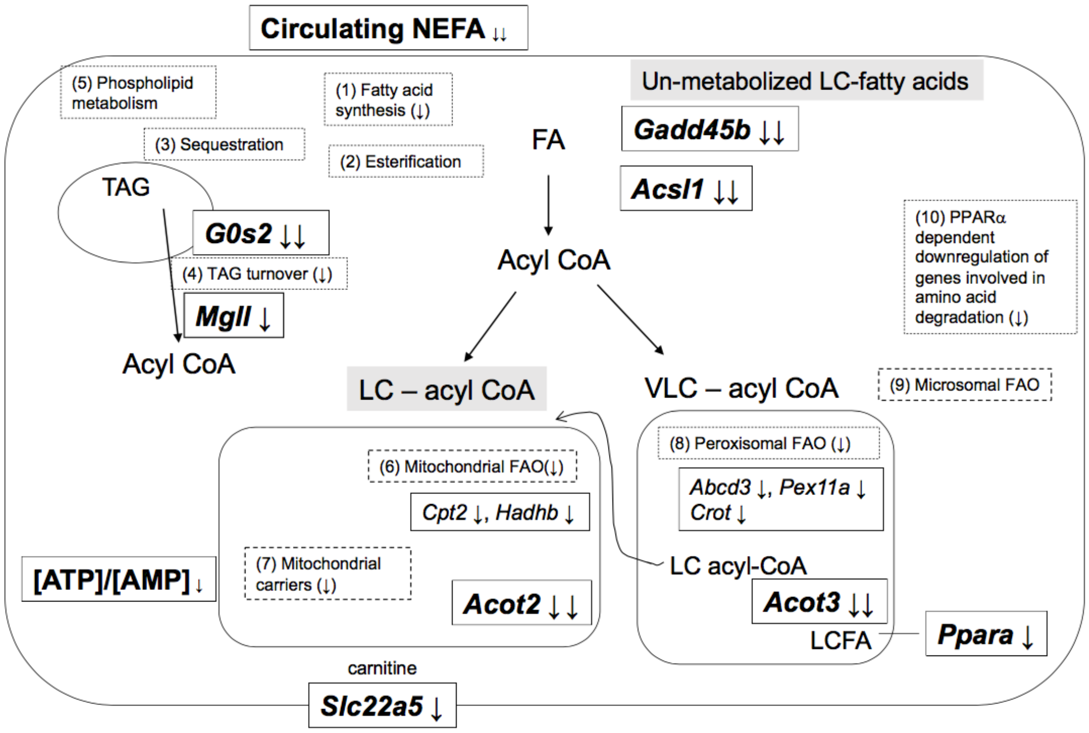

b. Expression of genes in PPARα dependent pathways related to lipid and amino acid metabolism

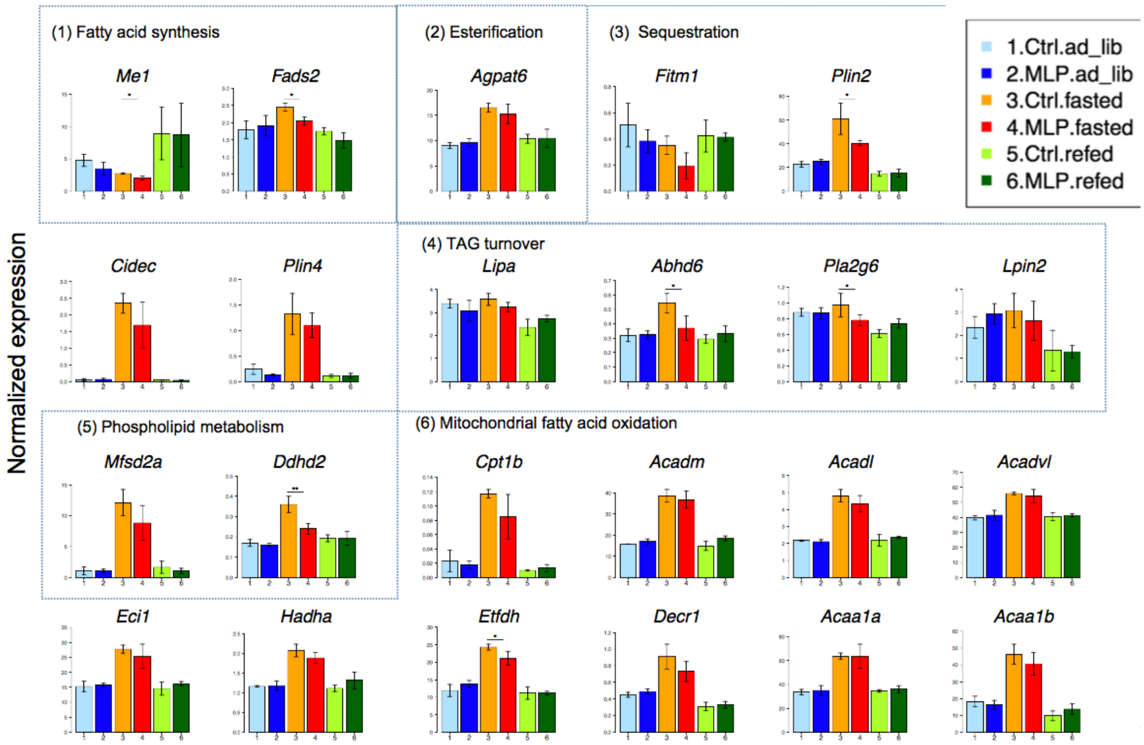

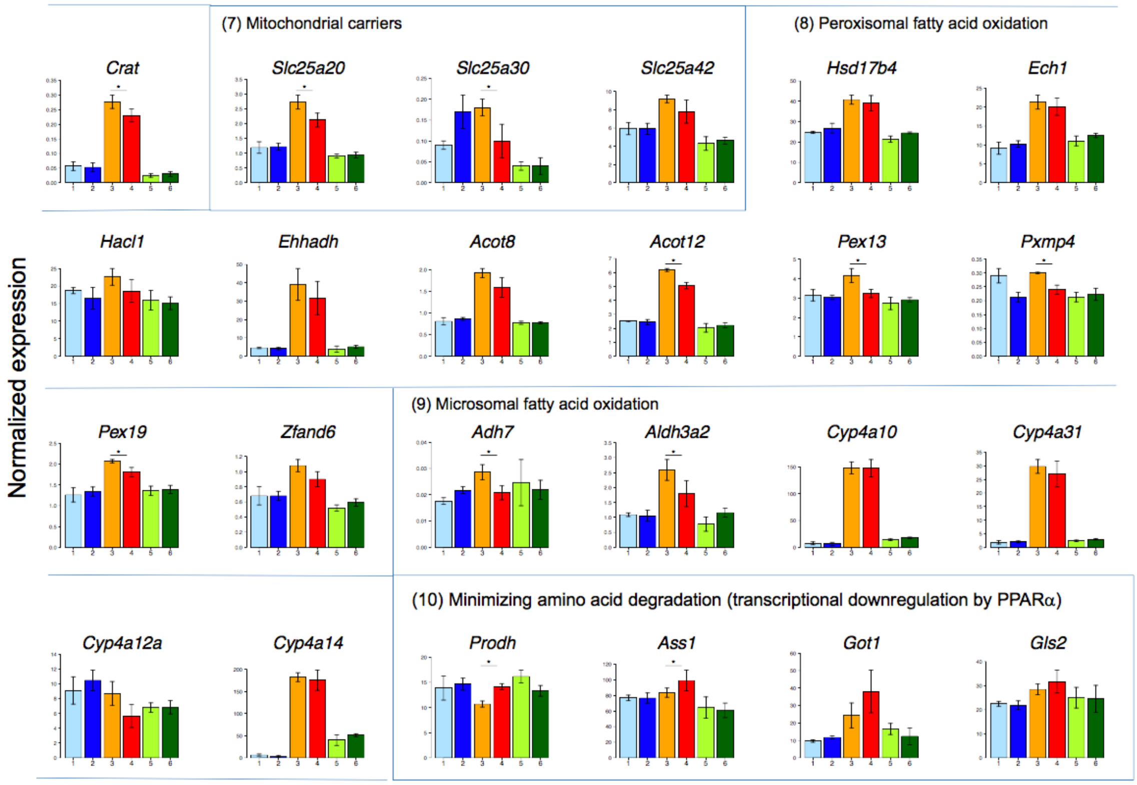

c. Expression of genes in other pathways regulated during a fasting-feeding cycle

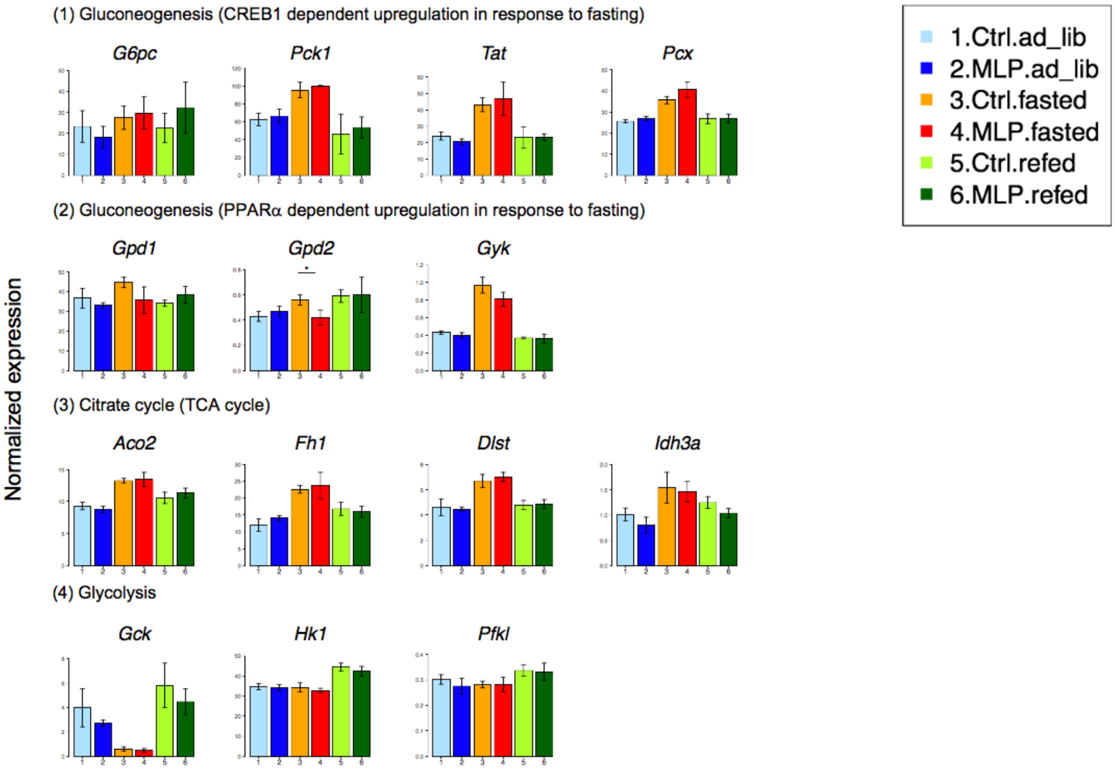

(5) Lipogenesis

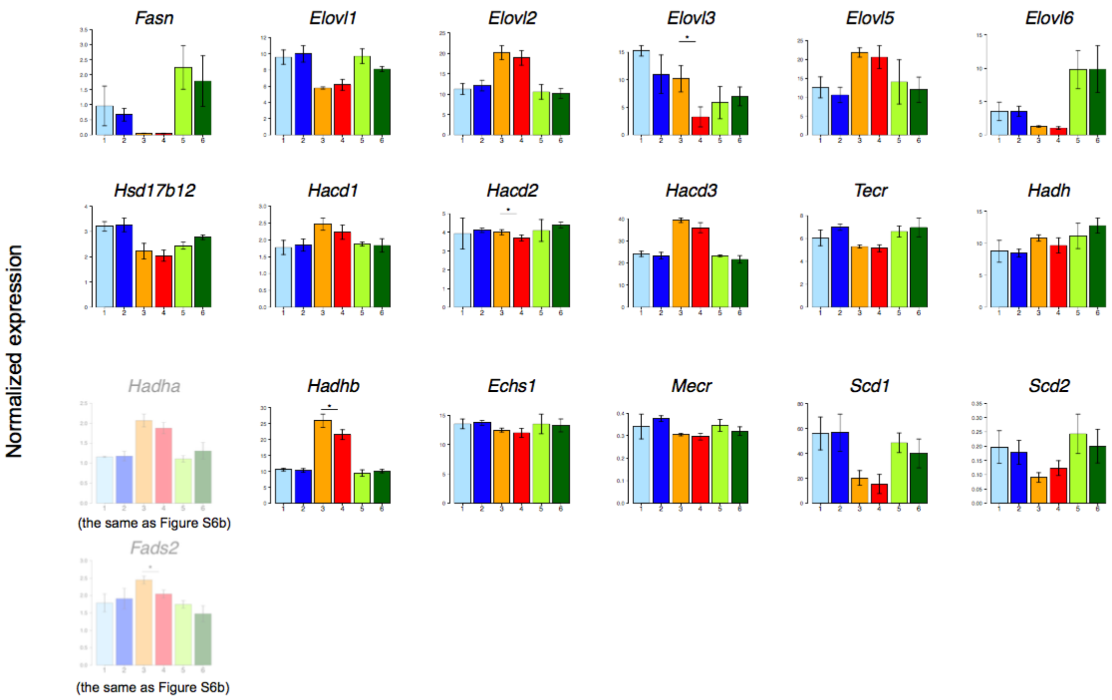

(6) Fasting responsive transcription factors (TFs)

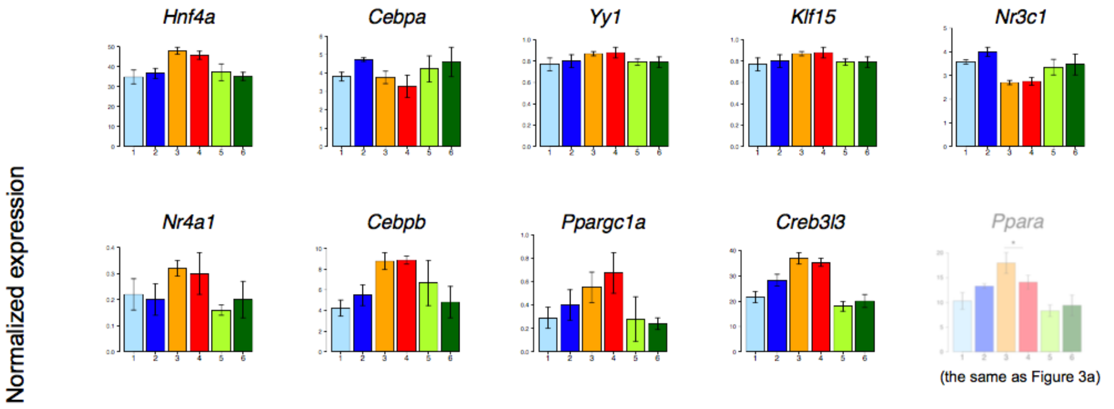

(7) PPARγ targets

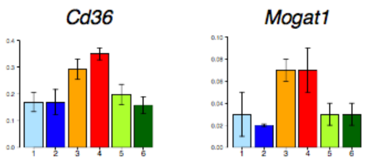

Supplementary Figure S6. **Microarray expression data for genes in response to fasting**

(a) Scheme summarizing the changes related to lipid metabolism observed in MLP pups at 24-h fasting.

Transcriptional and metabolic controls related to lipid metabolism were aberrantly regulated in the fasted state in the offspring exposed to early gestational maternal low protein (MLP) diet. The fasting-dependent induction of the listed genes were significantly impaired in the MLP pups, as described in the main text. The levels of circulating nonesterified fatty acids (NEFA) were decreased probably due to the reduced induction of *Gadd45b*[16]. The upregulation of *Ppara* as well as the genes involved in producing PPARα ligands (*Acsl1, Acot2/3/4*) were suppressed in the MLP pups. It is known that circulating NEFA cannot activate PPARα[22]. As shown in (b), most of the genes in PPARα activated pathways were uniformly downregulated in the MLP pups. The categorical terms (1-10) of the PPARα activated pathways refers and follows to the terms listed in the reference papers[22,36].

(b) Expression of genes in PPARα dependent pathways related to lipid and amino acids metabolism. (c) Expression of genes in other pathways regulated during a fasting-feeding cycle. (b and c) The microarray expression data for the representative genes involved in PPARα dependent pathways and fasting-refeeding response pathways are shown. Values were75 % tile-normalized. Data represent mean ± SD. (N = 3-4 animals/group) *P < 0.05; two-tailed Student's t-test. **FDR < 0.1; Benjamini and Hochberg's method.

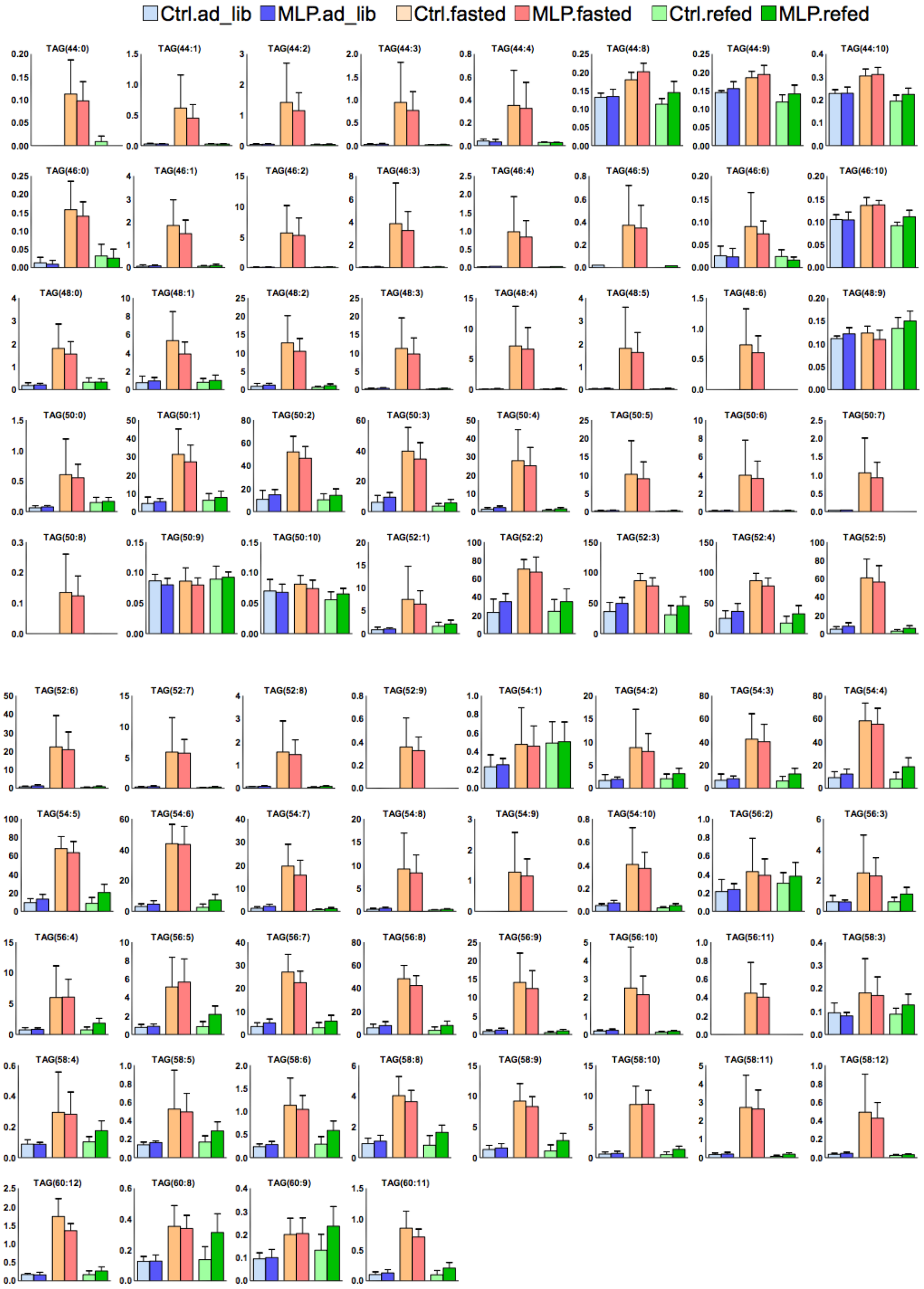
Ctrl.ad_lib
MLP.ad_lib
Ctrl.fasted
MLP.fasted
Ctrl.refed
MLP.refed
TAG(44:0)
TAG(44:1)
TAG(44:2)
TAG(44:3)
TAG(44:4)
TAG(44:8)
TAG(44:9)
TAG(44:10)
TAG(46:0)
TAG(46:1)
TAG(46:2)
TAG(46:3)
TAG(46:4)
TAG(46:5)
TAG(46:6)
TAG(46:10)
TAG(48:0)
TAG(48:1)
TAG(48:2)
TAG(48:3)
TAG(48:4)
TAG(48:5)
TAG(48:6)
TAG(48:9)
TAG(50:0)
TAG(50:1)
TAG(50:2)
TAG(50:3)
TAG(50:4)
TAG(50:5)
TAG(50:6)
TAG(50:7)
TAG(50:8)
TAG(50:9)
TAG(50:10)
TAG(52:1)
TAG(52:2)
TAG(52:3)
TAG(52:4)
TAG(52:5)
TAG(52:6)
TAG(52:7)
TAG(52:8)
TAG(52:9)
TAG(54:1)
TAG(54:2)
TAG(54:3)
TAG(54:4)
TAG(54:5)
TAG(54:6)
TAG(54:7)
TAG(54:8)
TAG(54:9)
TAG(54:10)
TAG(56:2)
TAG(56:3)
TAG(56:4)
TAG(56:5)
TAG(56:7)
TAG(56:8)
TAG(56:9)
TAG(56:10)
TAG(56:11)
TAG(58:3)
TAG(58:4)
TAG(58:5)
TAG(58:6)
TAG(58:8)
TAG(58:9)
TAG(58:10)
TAG(58:11)
TAG(58:12)
TAG(60:12)
TAG(60:8)
TAG(60:9)
TAG(60:11)

Supplementary Figure S7. Changes in hepatic triacylglycerol (TAG) composition by maternal diet and nutritional conditions.

Hepatic TAG metabolism in the control for (Ctrl.ad_lib) ad libitum, (Ctrl.fasted) fasted, and (Ctrl.refed) refed states, and in the MLP diet for (MLP.ad_lib) ad libitum, (MLP.fasted) fasted, and (MLP.refed) refed states. Total TAG fatty acid carbon chain length is shown in each figure. Y axis indicates the ratio of the peak area of the corresponding metabolite to that of internal standard, SM (d18:1/12:0). All data are shown as mean ± SD. Control ad lib, N = 8; MLP ad lib, N = 10; control fasted, N = 8; MLP fasted, N = 9; control refed, N = 8; MLP refed, N = 10. MLP, maternal low protein.

Dataset 1. Reference gene expression datasets

Dataset 2. 655 upregulated genes by 24-h fasting

Dataset 3. 781 downregulated genes by 24-h fasting

Dataset 4. PPARα-inducible genes and functional terms
